# Exploring influences of past learning experiences, individualist-collectivist cultural identity and social value orientations on Chinese and UK undergraduates' learning preferences

**DOI:** 10.3389/fpsyg.2025.1555675

**Published:** 2025-06-20

**Authors:** Chengcheng Ma, Shelley McKeown, Jo Rose

**Affiliations:** ^1^Institute for Intelligent Society Governance, Tsinghua University, Beijing, China; ^2^School of Education, University of Bristol, Bristol, United Kingdom; ^3^Department of Experimental Psychology, University of Oxford, Oxford, United Kingdom

**Keywords:** learning preferences, social value orientation, individualist-collectivist culture, learning experiences, cooperative learning

## Abstract

**Introduction:**

The increasing enrolment of Chinese students in UK higher education (HE) has brought various challenges, particularly the difficulties they encounter in adapting to the Western classroom environment. While previous studies often considered Chinese students' learning preferences as culturally determined, such portrayals risk oversimplification and neglect the individual variations in educational and cultural experiences. Recognizing the necessity of understanding the learning preferences of both Chinese and UK students, this study seeks to move beyond simplistic cultural characterizations. Specifically, this study examines the influences of Chinese and UK university students' past learning experiences, individualist-collectivist (I-C) cultural identity, and individual characters of social value orientation (SVO) on their preferences for cooperative, competitive and individualistic learning approaches.

**Methods:**

A total of 562 undergraduates completed an online questionnaire assessing these constructs.

**Results:**

Structural equation modeling showed that cooperative learning experiences were positively associated with cooperative learning preferences, and negatively with individualistic learning preferences. Competitive learning experiences were positively related to competitive learning preferences. SVO was positively related to I-C cultural identity and cooperative learning preferences. I-C cultural identity was found to mediate the paths between SVO and competitive and individualistic learning preferences. Furthermore, the multigroup analysis revealed that these relationships were different in the UK and Chinese undergraduates.

**Discussion:**

Current findings highlight the complex interplay of educational and cultural factors and individual characters in shaping learning preferences. The study provides valuable insights for creating inclusive and culturally responsive learning environments in HE.

## 1 Introduction

In recent years, the globalization of HE has continued to accelerate, marked by a sustained increase in international student mobility. According to Higher Education Statistics Agency (2022), in the academic year 2021/22, more than 150,000 Chinese students were enrolled in the UK HE, representing approximately one-third of non-European Union students in the UK. China is now the top sending country for international students in the UK. This trend not only reshapes educational landscapes but also foregrounds the critical role of social interactions in learning environments. For example, the “silent Chinese students” issue, characterized by hesitancy in class participation and a preference for independent work, often poses a significant concern for educators, teachers and practitioners (Ping, 2010). Understanding how cultural and social dynamics influence learning within these diverse settings is crucial for adapting educational practices to the needs of a global student body. The current study narrows its focus to the investigation of learning preferences among Chinese and UK home undergraduates.

Prior studies often depict Chinese students' learning preferences as predominantly influenced by cultural norms, such simplifications overlook the diversity and the dynamic nature of social interactions that shape their perceptions of and preferences for learning environment (Gill, 2007). Indeed, educational experiences regarding the varying pedagogical practices and learning environments that exist across different countries and cultures play a pivotal role in shaping students' learning preferences and perceptions of learning contexts (Campbell and Li, 2008; Qu and Cross, 2024). For example, Chinese students were often exposed to an educational ethos characterized by high-stakes examinations and a competitive learning environment (Clark et al., 2007), shaping a unique set of learning preferences focused on individual achievement. In contrast, UK students typically experienced a more discussion-based and collaborative educational setting (OECD, 2014). Hence, the way Chinese and UK students relate to others as part of their learning preferences is shaped by their experienced social-cultural and educational environment. These distinct backgrounds provide a rich context for exploring how different educational cultures influence student learning preferences.

Recent studies have begun to investigate the complexities of learning preferences in a cross-cultural context, highlighting the influence of cultural and educational systems on student engagement and preference formation (Elliot et al., 2016; Nguyen-Phuong-Mai, 2019; Qu and Cross, 2024). However, a direct comparison between Chinese and UK students remains scarce. Understanding the distinct cultural and educational experiences of Chinese and UK students is essential for educators and practitioners. Furthermore, this understanding should extend beyond national-level generalizations to recognize the unique character of each student. It is crucial to emphasize that while acknowledging these macro-level differences, the significance of investigating these influences at the individual level should not be overshadowed, as previous studies have shown that intra-cultural variability significantly shapes individual behaviors and learning preferences (Wagner, 1995; Taras et al., 2010).

While previous studies have explored learning preferences in isolated cultural contexts, there remains a lack of research that examines the interplay between past learning experiences, I-C cultural identity, and SVOs in shaping learning preferences across distinct cultural settings. Drawing on social interdependence theory (Johnson and Johnson, 2005) and the I-C cultural framework (Triandis, 2001), we argue that these constructs represent different types of influences. Specifically, past learning experiences reflect situational influences, I-C cultural identity reflects cultural influences, and SVOs reflect dispositional or personality-based influences. Examining their interplay allows us to develop a holistic and comprehensive understanding of how educational, cultural and individual factors together shape students' learning preferences across different cultural settings.

To address this gap, this study conducts a comparative analysis between Chinese and UK undergraduates, providing insights into how these factors interact within different educational and cultural contexts. By doing so, we seek to move beyond stereotypes, recognizing the diversity within each student cohort. This study contributes to the understanding of learning preferences while respecting the diversity within student populations from different cultural and educational backgrounds. Findings are expected to contribute significantly to the creation of inclusive and culturally responsive educational environments.

## 2 Literature review

### 2.1 Learning preferences

Students may have individual differences in preferences for cooperative, competitive, or individualistic learning (Elliot et al., 2016; Gocłowska et al., 2017; Johnson and Johnson, 2005). Students with cooperative learning preferences often enjoy learning collaboratively with their peers through knowledge sharing and mutual support and tend to achieve both their own and others' learning goals (Johnson and Johnson, 2009; Grasha, 2002). Conversely, students with a competitive learning preference tend to compete with their peers to achieve learning goals and maximize their own learning outcomes relative to others—they often treat learning as a competition with only a few winners and many losers (Montgomery and Groat, 1998; Johnson and Johnson, 2005). Students with individualistic learning preferences tend to avoid interacting with their peers and focus on their own learning outcomes, with little regard for others (Gocłowska et al., 2017; Johnson and Johnson, 2005).

Learning preferences can influence how individuals perceive learning situations and the appropriate actions they should take to interact with peers, which play an essential role in the effectiveness of learning (Gocłowska et al., 2017; Johnson and Johnson, 2009; Owens and Barnes, 1982). With a better understanding of students' learning preferences would help educators to enhance teaching strategies and improve student outcomes.

### 2.2 Factors influencing learning preferences

#### 2.2.1 Past learning experiences

Students' past learning experiences play a pivotal role in shaping their learning preferences. Johnson and Johnson (2009) argued that students' value system regarding cooperation, competition and individualistic actions can be learned in their school lives. According to social interdependence theory (Deutsch, 1949, 1962), social interdependence occurs when individuals' outcomes are interdependently influenced by the actions of others, which explains how people's cooperative, competitive, or individualistic behaviors are interdependently influenced by situational factors (including others' behaviors). Johnson and Johnson (2005) extended the social interdependence theory and argued that students' learning preferences are influenced by the learning environment with different interdependent structures (i.e., cooperative, competitive, individualistic structure). Thus, students' learning preferences are at least partly shaped and sharpened by their learning experiences, in other words their history of interacting with others in learning situations over time (Choi et al., 2011; Johnson and Johnson, 2005, 2009).

Students frequently engaging in cooperative learning often consider learning outcomes beneficial to themselves and others and tend to believe that all group members will sink or swim together (Johnson and Johnson, 2005). In a cooperative learning situation, students are encouraged to exchange knowledge and skills; this could, in turn, facilitate students' positive interdependence and individual accountability (Slavin, 2014). When positive interdependence exists in a learning environment (i.e., a cooperative learning environment), students tend to work together in small groups to maximize all the group members' learning outcomes mutually. For instance, students may share their materials and support each other. Substantial evidence shows that cooperative learning contributes to developing mutual concern and interpersonal trust among students, promoting students' cooperative learning preferences (Kochanska, 2002).

Conversely, when repeatedly engaging in competitive learning activities, students may strive to work faster and more accurately to outperform their classmates (Johnson and Johnson, 2005), which in turn reinforces their competitiveness. Competitive learning creates a situation where students need to achieve learning goals in a better manner than their peers (Montgomery and Groat, 1998). Having more experiences of competitive learning can enhance competitiveness and individualistic intention, which could promote competitive and/or individualistic learning preferences (Johnson and Johnson, 2009).

#### 2.2.2 The role of I-C culture

Recent studies have increasingly highlighted the complex role culture plays in shaping students' learning preferences. The I-C cultural dimension remains a crucial factor in understanding these preferences. For instance, Hofstede et al. (2010) and Triandis (2001) have extensively discussed how collectivist cultures, like China, emphasize group interests and interdependence, whereas individualist cultures, like the UK, value independence and self-autonomy. More recent studies, such as Elliot et al. (2016), Pan and Cutumisu (2024), and Zhang and Han (2023), have further explored how cultural dimensions impact learning behaviors and preferences across different societies. China is often characterized as collectivist culture (Chen-Xia et al., 2023; Hofstede et al., 2010; Zhang and Han, 2023). Collectivist students tend to prefer group working and have better performance in groups (Biggs, 1991; Hofstede et al., 2010). The mentality of collectivist culture value supports in-group cooperation and promote collective success (Nguyen et al., 2006; Nguyen-Phuong-Mai, 2019), which contributes to preference for cooperative learning (Trompenaars, 1993). Unlike China, the UK is often characterized by an individualist culture (Chen-Xia et al., 2023; Hofstede et al., 2010). In theory, the core assumption of individualist culture is that people are independent and focus on own needs and uniqueness, instead of prioritizing the collective (Hofstede et al., 2010; Triandis, 2001). Arguably, the character of individualism seems to reject the principle at the center of cooperative learning: that the learning goals of each student can only be achieved if all group members achieve their learning goals (Johnson and Johnson, 2005). Thus, one can infer that individualist culture tend to be associated with competitive or individualistic learning preference.

Although the large culture is often considered as a critical factor relevant to learning preferences, it should be acknowledged that Chinese and UK students' culture value cannot be over-generalized by national cultural character. In other words, only employing the large culture perspective could not be capable to capture the individual variation within culture. Wagner (1995) argued that variations in individuals' personal endorsement to I–C cultural value affects their preferences toward cooperating or competing in group situations, signifying how fundamental differences between personal endorsement to I-C cultural values can affect learning preferences. In this study, to understand the I-C culture influence on Chinese and UK undergraduates' learning preferences at an individual level, this study focus on Chinese and UK students' I-C cultural identity—an individual's sense of belonging and identification with individualist or collectivist culture. Cultural identity affects individuals' perceptions of what is valued and appropriate within their cultural context. Thus, students may have learning preference that align with their cultural identity to maintain a sense of identity and social cohesion. Previous studies have shown that I-C cultural identity was found to predict peoples' cooperation related inclinations (Wagner, 1995; Lampridis and Papastylianou, 2017). We, therefore, hypothesize that students' I-C cultural identity may influence learning preferences.

#### 2.2.3 Individual character of SVO

In addition to the influence of educational and cultural experiences, students' individual character of value orientation toward cooperation, competition and individualistic efforts can also affect learning preferences. Compared to other personal individual character (e.g., extroversion/introversion), SVO is a relatively distinct trait related to cooperation and competition (Kurzban and Houser, 2001). SVOs, in most literature, can be defined as a relatively “stable personality trait” that can reflect people's preference for resource allocation, and further predicts individuals' cooperative, competitive, and individualistic inclinations and actions in different contexts (McClintock, 1978; Messick and McClintock, 1968; Smeesters et al., 2003). Two types of SVOs are generally identified: prosocial and proself (Bogaert et al., 2008; Smeesters et al., 2003). People with prosocial value orientation tend to cooperate with others and seek win-win solutions to disagreements (Smeesters et al., 2003; Van Lange et al., 2007). In contrast, proselfs have a default behavior of striving for self-interest, with goals of achieving a relative advantage over others (competitive value orientation) or maximizing their own interest (individualistic value orientation). As an important interpersonal orientation and tendency that drives social interactions, SVOs can have considerable implications for understanding cooperative, competitive and individualistic learning preferences.

Prosocials are regarded as sensitive to information signaling trustworthiness and cooperation; this could, in turn, substantiate an expectation that their cooperative or prosocial behaviors (e.g., helping or sharing) are reciprocated (Bogaert et al., 2008). Cooperative learning encourages students to engage in cooperation and learn through a mutually beneficial approach and promotes inter-student trustworthiness (Johnson and Johnson, 2009), which seems to align with a prosocial orientation. Competitive learning meets the inherent goals of competitors since it provides a platform to stimulate students to compete for a relatively higher grade or rank (Johnson and Johnson, 2005). Individualistic learning requires students to learn independently of each other and for themselves (Johnson and Johnson, 2005), resonating with individualistic value orientations. We hypothesize that students' individual differences in SVOs could account for potential variations in learning preferences.

#### 2.2.4 The interplay between SVOs and I-C cultural identity

Emerging research highlights the interplay between individual value orientations and cultural identity in shaping behavioral tendencies and preferences, including learning preferences. SVO has been shown to play a critical role in predicting personal cultural tendencies and the endorsement of certain cultural values, with these relationships varying across societies (Moon et al., 2018).

Drawing from social interdependence theory (Deutsch, 1949, 1962) and the individualist-collectivist cultural framework (Hofstede et al., 2010; Triandis, 2001), cultural identity is posited as a lens through which SVOs influence learning preferences. Specifically, a collectivist cultural identity may amplify prosocial value orientation, fostering cooperative learning preferences. Conversely, an individualist identity may alight with proself orientations, promoting competitive or individualistic preferences.

These dynamics suggest that cultural identity serves as a mediating factor through which SVO influence learning preferences, as supported by prior findings (e.g., Lampridis and Papastylianou, 2017; Van Lange et al., 2007). SVOs do not act independently of cultural context, and their influences can vary depending on how individuals internalize cultural norms and values (Moon et al., 2018). For instance, people with prosocial value orientation may align with cooperative learning preferences in collectivist contexts but could be interpreted differently in individualist settings. Therefore, by examining the interplay between I-C cultural identity and SVOs, we can gain a fuller picture of how personal dispositions and cultural affiliations may jointly shape learning preferences. Few studies, however, have explicitly examined the interplay between SVOs and I-C cultural identity in shaping learning preferences, particularly in cross-cultural contexts like China and the UK. This study aims to address this gap by providing a comprehensive understanding of how cultural and individual factors jointly influence learning behaviors, offering insights into cooperative, competitive, and individualistic learning preferences.

### 2.3 Research questions and hypotheses

Despite the growing body of research on learning preferences, few empirical studies have examined the factors shaping these preferences, particularly through a cross-cultural lens comparing the UK and China.

China and the UK represent two distinctive paradigms in higher education, shaped by different cultural values and pedagogical practices. China's high-stakes, exam-oriented system fosters a competitive ethos within a collectivist cultural framework, where students' success is often tied to familial and societal expectations. The UK, however, emphasizes student-centered, discussion-based, and collaborative learning practices that align with individualist values of self-autonomy and critical thinking. These differences offer a compelling theoretical basis for cross-cultural investigation.

This research holds practical significance for educators in globalized higher education. Chinese students represent the largest international cohort in UK universities, therefore, understanding how their past learning experiences and cultural identity influence adaptations to UK classrooms is essential. Furthemore, as UK classrooms become increasingly diverse, insights from this study inform the development of culturally responsive teaching strategies that bridge the gap between competitive and cooperative learning preferences, contributing to creating an inclusive and collaborative education environment.

**Figure 1:**
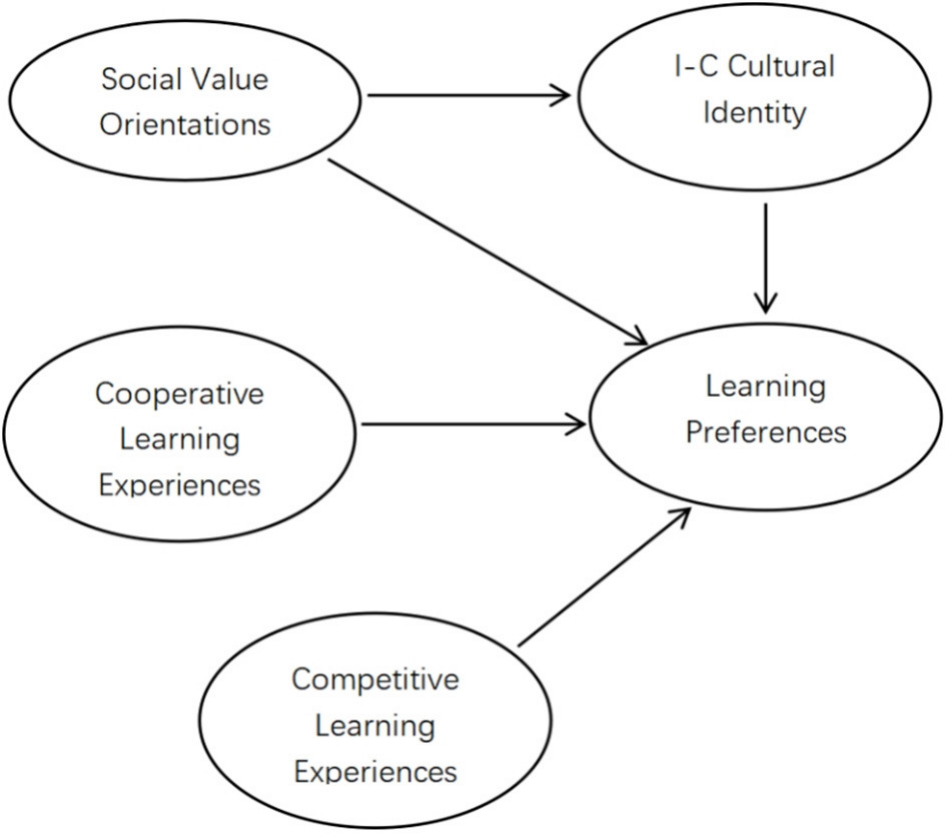
The hypothesized relationship between learning experiences, social value orientations, individualist-collectivist cultural identity, and learning preferences.

While recent studies (e.g., Qu and Cross, 2024; Pan and Cutumisu, 2024) have explored cross-cultural learning preferences, comprehensive models that integrate past learning experiences, I-C cultural identity, and SVOs remain scarce. To address this gap, our study investigates the relationships between past learning experiences, I-C cultural identity, SVOs and learning preferences focuses on UK and Chinese undergraduates. Based on the literature, we propose a hypothesized model (see Figure 1).

Drawing on previous research literature, it is hypothesized that:

**H1: Influence of past learning experiences on learning preferences**

**H1a**: Past cooperative learning experiences are positively associated with cooperative learning preferences, yet negatively with competitive learning preferences and individualistic learning preferences.
**H1b**: Conversely, past competitive learning experiences are positively associated with competitive learning preferences and individualistic learning preferences, yet negatively with cooperative learning preferences.

**H2: Influence of I-C cultural identity on learning preferences**

Higher levels of collectivist cultural identity are positively associated with cooperative learning preferences, yet negatively with competitive learning preferences and individualistic learning preferences.

**H3: Influence of SVOs on learning preferences**

Higher levels of prosocial value orientation are positively associated with cooperative learning preferences, yet negatively with competitive learning preferences and individualistic learning preferences.

**H4: Mediating role of I-C cultural identity**

I–C identity mediates the relationship between SVO and learning preferences, including cooperative, competitive, and individualistic learning preferences.

I–C cultural identity and SVO are conceptualized as continuous constructs, where higher scores indicate stronger collectivist and prosocial orientations, respectively. As such, effects related to individualist or proself orientations are interpreted as inverse relationships of those specified above. This avoids redundancy and reflects standard practice in using continuous latent variables.

In addition, we propose that these relationships may differ between Chinese and UK participants, due to the difference in socio-cultural contexts between the two countries.

## 3 Method

### 3.1 Participants

Chinese participants were recruited from a research-intensive university located in East China, and UK participants were recruited from two research-intensive universities in the UK. We focused specifically on undergraduate students aged 18 to 23, as prior research (Van Lange et al., 1997) suggests that SVOs in this population are less affected by chronological age compared to postgraduate students. This age criterion also allowed to reduce potential confounding influences stemming from diverse life experiences. Additionally, by recruiting domestic Chinese and UK undergraduates, we aimed to minimize the impact of prior cross-cultural living experiences that could have independently shaped participants' learning preferences.

Participants were sent the survey link either from their faculty staff and faculty online survey system. The final Chinese sample comprised 260 undergraduates (72 male and 188 female) with a mean age of 20.7 (*SD* = 1.29), and the final UK sample comprised 302 undergraduates (56 male and 246 female) with a mean age of 18.9 (*SD* = 0.96).

### 3.2 Sample sizes and power calculation

To meet the sample size recommendation in our analysis method (i.e., PLS-SEM) for a statistical power of 80%, we follow the guidance developed by Cohen (2016) and Hair et al. (2017). Each of these subpopulations exceeds the minimum for the theoretical model that has four arrows pointing at a construct—i.e., learning preferences (10% with a minimum *R*^2^ of 0.10 = 111). In order to exceed the minimum *R*^2^ of 0.1 at a 5% significance level, both the Chinese and UK subgroup s would need to exceed 137 (Hair et al., 2017). Ultimately, a subsample of 191 for both Chinese and UK groups would provide a significance level of.1%. In this study, the sample size for the Chinese subgroup is 260 and for the UK subgroup is 302, which meets the minimum sample size criteria.

While the sample size exceeds the minimum requirements for PLS-SEM analysis, it is important to note that the sample may not fully represent the diverse student populations in both countries. Future studies should consider more diverse sampling strategies to enhance generalizability.

### 3.3 Materials and procedure

Prior to data collection, ethical approval was obtained. Data were collected through online survey, using “Wenjuanxing” for Chinese participants and “Qualtrics” for UK participants. In each context, the same measures were included in an online survey, including participants' demographics, past learning experiences, I-C cultural identity, learning preferences and SVOs. Measures for Chinese participants were translated and back-translated from English to Mandarin.

Among these constructs, participants' previous learning experiences, I-C cultural identity, learning preferences were assessed through self-reported data using established scales (see blow in order), while SVOs were examined using behavioral economics games.

#### 3.3.1 Previous learning experiences

We mainly focus on two types of learning experiences—cooperative vs. competitive. Individualistic learning experiences were not examined separately because individualistic learning behaviors are often embedded within both cooperative and competitive learning environments, making them less distinguishable as a standalone experiential category. While students may engage in individual work in either setting, such experiences do not necessarily reflect a consistent or contextually distinct learning mode. Nevertheless, we retained individualistic learning preferences as a dependent variable to capture students' dispositional tendency to engage in tasks independently, which may be shaped by broader cultural, personal, or situational factors. Previous cooperative learning experiences were measured using eight items adapted from the What is Happening in this Class questionnaire (WIHIC; Fraser, 1998), such as “*I got along with other students when doing assignment work”* (α = 0.88 China, α = 0.81 UK). Previous competitive learning experiences were measured by seven items revised from the Learning Environment Inventory (LEI, Fraser et al., 1982), such as “*Most students wanted their work to be better than their friends' work*” (α = 0.68 China, α = 0.83 UK).

#### 3.3.2 I–C cultural identity

Participants' I–C cultural identity was measured using 20 items with a 7-point Likert scale from Wagner (1995). Sample items include, “*A group is most efficient when its members do what they think is best rather than doing what the group wants them to do*”. This measure comprises five dimensions: Stand Alone, Win Above All, Group Preference, Sacrifice in Group, and Individual Thinking. Prior studies confirmed that these dimensions load sufficiently onto a single higher-order construct, justifying their aggregation (Wagner, 1995). This approach aligns with the theoretical premise that individual cultural identity has multiple facets of individualism and collectivism. Cronbach's alphas for each dimension of I–C cultural identity scale was as follows: Stand Alone (five items, α = 0.80 China, α = 0.77 UK); Win Above All (five items, α = 0.84 China, α = 0.76 UK); Group Preference (three items, α = 0.58 China, α = 0.87 UK); Sacrifice in Group (four items, α = 0.84 China, α = 0.79 UK); and Individual Thinking (three items, α = 0.85 China, α = 0.80 UK).

Although Wagner originally conceptualized I–C identity as multidimensional, his validation study supports the interpretation of a single higher-order factor. We therefore treated I–C cultural identity as a composite construct. This approach aligns with our theoretical focus on the broader influence of cultural orientation rather than the unique effects of its subdimensions. Practically, using a unidimensional construct enhances parsimony and facilitates comparison across groups in a structural equation modeling (PLS-SEM) framework. This approach is also supported by recent studies that have adopted the composite score from Wagner's measure to capture an overall I-C cultural identity (e.g., Wen et al., 2023; Zhang et al., 2024).

#### 3.3.3 Learning preferences

Learning preferences were measured by the social interdependence scale (Johnson and Norem-Hebeisen, 1979; Choi et al., 2011), which consists of 22 items with a 7-point Likert design (*strongly disagree* to *strongly agree*). Seven items measure students' cooperative learning preference (α = 0.92 China, α = 0.90 UK), for example “*I like to share my ideas and materials with other students”*. Eight items measure competitive learning preference (α = 0.89 China, α = 0.88 UK), for example “*I work to get better grades than other students do”*. Seven items measure individualistic learning preference (α = 0.79 China, α = 0.91 UK), for example “*I work better when I work alone”*.

#### 3.3.4 SVO

In addition to the above self-reported measures, to mitigate the potential biases associated with self-reported data and to gain an accurate understanding of participants' SVOs, we employed behavioral economics games, specifically the SVO slider measure (Murphy et al., 2011) into our online survey.

Unlike traditional self-report instruments, the SVO slider measure employs a decision-making format that avoids leading questions and helps minimize social desirability effects, providing a more behaviorally grounded view of participants' tendencies toward prosocial, individualistic, or competitive actions (Murphy and Ackermann, 2014).

Participants were incentivised with corresponding monetary rewards based on their decision-making results. This is because participants may not have an incentive to admit their true social preferences when it costs nothing to pass for being cooperative and prosocial. Using behavioral economics experiment, however, can contribute to observing participants' actual behaviors rather than intentions (Gächter et al., 2010).

In this measure, participants make a decision about allocating resources (e.g., money) between themselves and another partner (someone the participants do not know). All 15 items in the measure are a resource allocation selection over a continuum of joint payoffs, with options varying according to how much is allocated to the self and to the other. Responses to this measure provide an integrated ranking of different value orientations.

**Figure 2:**
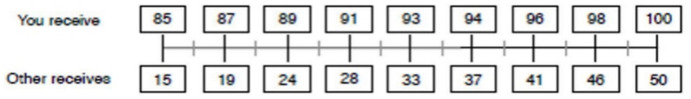
Example 1.

In the first example (see Figure 2), a specific choice of joint payoff allocation of “*you receive 100; the other receives 50”* refers to a choice to maximize personal gains, suggesting an individualistic value orientation. In contrast, a joint payoff allocation of “*you receive 85; the other receives 15”* represents a choice of maximizing the relative difference between one's own and one's partner's gains in favor of self-interest, which suggests a competitive value orientation.

**Figure 3:**
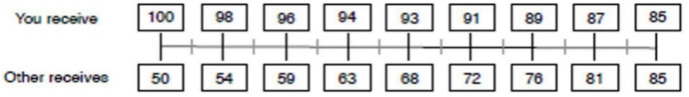
Example 2.

In the second example (see Figure 3), a joint payoff allocation of “*you receive 85; the other receives 85”* represents a choice of maximizing joint gains, which indicates a prosocial value orientation. Conversely, a joint payoff allocation of “*you receive 100; the other receives 50”* reflects a choice of maximizing the relative difference between one's own and one's partner's outcome, suggesting a competitive value orientation.

Participants were informed they would have a chance of winning a monetary reward (randomly selected by the computer), and this reward was dependent on their choices of the SVO slider measure. Incentives thus followed the random lottery incentive system, which avoids problems associated with other incentive schemes (Lee, 2008) and has been shown to elicit behavior in line with true preferences (Cubitt et al., 1998). The actual amount of money each of the Chinese and UK participants had a chance to win was about 100 yuan (approximately equal to £10) and £10, respectively, which was available for five participants in each group.

The use of this incentive-compatible behavioral measure is well-established in experimental economics and has been increasingly applied in cross-cultural psychological research (e.g., Gächter et al., 2010). It has also been validated and employed in studies conducted in the Chinese context (e.g., Ma and Shen, 2024). The measure enables a more objective understanding of participants' social preferences beyond self-perceptions, which is particularly important in studies examining culturally sensitive constructs like prosociality and competitiveness.

## 4 Results

After the data were collected, they were downloaded into SPSS and analyzed in SmartPLS (Ringle et al., 2023). There was no missing or incomplete data, however, two Chinese and one UK participants' responses were excluded from the final analyzed sample: one Chinese participant's age did not meet our sampling requirement (age = 46 years, which is over 23 years old); and two participants' submitted responses included frequent extreme scores (Field, 2013).

### 4.1 Descriptive data analyses

Correlations between previous learning experiences, SVO, I-C cultural identity, and learning preferences were examined separately for Chinese and UK groups (see Table 1).

**Table 1:** Correlations of previous learning experiences, SVOs, I-C cultural identity, and learning preferences.

**Variable**	**CLE**	**CME**	**I–C**	**CLP**	**CMP**	**INP**	**SVO**
CLE	—	0.025	0.184^**^	0.368^**^	−0.118^*^	−0.426^**^	−0.043
CME	0.357^**^	—	−0.175^**^	−0.003	0.328^**^	0.046	−0.148^*^
I–C	−0.078	−0.273^**^	—	0.359^**^	−0.421^**^	−0.506^**^	0.200^**^
CLP	0.661^**^	0.325^**^	−0.015	—	0.415^**^	−0.279^**^	0.076
CMP	0.319^**^	0.487^**^	−0.440^**^	0.415^**^	—	0.257^**^	−0.132^*^
INP	−0.240^**^	0.174^**^	−0.551^**^	−0.279^**^	0.257^**^	—	−0.220^**^
SVO	−0.070	−0.176^**^	0.237^**^	0.076	−0.132^*^	−0.220^**^	—

* Correlation is significant at the 0.05 level (2-tailed).

** Correlation is significant at the 0.01 level (2-tailed).

CLE, cooperative learning experiences; CME, competitive learning experiences; SVO, social value orientation; I-C, I–C cultural identity; CLP, cooperative learning preference; CMP, competitive learning preference; INP, individualistic learning preference. Below diagonal = Chinese sample; Above diagonal = UK sample.

### 4.2 Analytical framework

First, we employ the partial least squares structural equation modeling (PLS-SEM) to analyse our combined data. PLS-SEM is a SEM approach that focuses on analyzing complex models with many variables, indicators, and structural paths, without imposing assumptions about the distribution of data (Hair et al., 2017). This approach is especially valuable for analyzing a theoretical framework from a predictive standpoint and for providing causal explanations (Sarstedt et al., 2016).

We then used multigroup analysis (MGA) PLS-SEM to estimate group-specific path coefficients to identify potential differences between UK and Chinese groups, aiming for explanations of observed heterogeneity and minimization of potential misrepresentation of results. We conduct MGA PLS-SEM and report the results based on the guidance of Hair et al. (2019).

#### 4.2.1 Assessment of measurement model

In this study, we followed PLS-SEM guidelines (Hair et al., 2017, 2019) to assess the measurement model and ensure the validity and reliability of translated and adapted measures across groups. Rather than employing traditional covariance-based CFA, PLS-SEM provides theoretically consistent alternative for evaluating construct validity through a combination of internal consistency reliability, convergent validity, discriminant validity, and measurement invariance. All reliability results exceeded the recommended value of 0.70 (Henseler et al., 2009), suggesting good internal reliability (see Table 2). As depicted in Table 2, Average Variance Extracted (AVE) of each construct surpassed the recommended value of 0.5, achieving convergent validity. All values of the Heterotrait-Monotrait Ratio (HTMT) ratio were < 0.85 or 0.90, indicating adequate discriminant validity (Henseler et al., 2015; see Table 3). Variance Inflation Factor (VIF) of each construct is below the threshold value of 3, indicating there was no issue of collinearity in this model.

**Table 2:** Construct reliability and validity.

**Variable**	**Cronbach's α**	**rho_a**	**rho_c**	**AVE**
CLE	0.835	0.840	0.875	0.501
CLP	0.875	0.876	0.915	0.730
CME	0.785	0.794	0.860	0.607
CMP	0.892	0.899	0.916	0.610
ICA	0.765	0.795	0.863	0.678
ICB	0.644	0.649	0.809	0.587
ICC	0.851	0.851	0.931	0.870
ICD	0.809	0.835	0.887	0.725
ICE	0.829	0.847	0.920	0.853
INP	0.884	0.884	0.915	0.684

CLE, cooperative learning experiences; CME, competitive learning experiences; ICA to ICE, each of the five dimensions of I–C cultural identity; CLP, cooperative learning preference; CMP, competitive learning preference; INP, individualistic learning preference.

**Table 3:** Heterotrait-monotrait ratio (HTMT)–matrix.

**Variable**	**CLE**	**CLP**	**CME**	**CMP**	**ICA**	**ICB**	**ICC**	**ICD**	**ICE**	**INP**
CLE	-									
CLP	0.622									
CME	0.212	0.132								
CMP	0.121	0.096	0.511							
ICA	0.122	0.127	0.323	0.580						
ICB	0.159	0.156	0.431	0.773	0.643					
ICC	0.444	0.336	0.192	0.193	0.099	0.175				
ICD	0.188	0.458	0.208	0.198	0.201	0.203	0.039			
ICE	0.138	0.171	0.303	0.414	0.537	0.422	0.399	0.129		
INP	0.245	0.219	0.169	0.300	0.295	0.476	0.573	0.173	0.111	
SVO	0.037	0.147	0.213	0.222	0.209	0.293	0.079	0.036	0.225	0.166

#### 4.2.2 Test for measurement invariance

We conducted a measurement invariance examination following the measurement invariance of composite models (MICOM) procedure (Henseler et al., 2016). Results showed that configural invariance, compositional invariance, and the quality of a composite's variance across groups are met, however, composites' mean values were different across Chinese and UK groups. Although full measurement invariance was not met, partial measurement invariance is confirmed, which allows us to compare the path coefficients with the MGA (Cheah et al., 2020).

#### 4.2.3 Assessment of structural model

After confirmation of acceptable reliability and validity of the measurement model and partial measurement invariance across Chinese and UK groups, structural model analysis was conducted with bootstrapping using 5000 samples to identify significant path coefficients. The *R*^2^ value (*R*^2^ = 0.45, 0.47, 0.46) indicates that 45, 47, and 45% of the variance in cooperative, competitive, and individualistic learning preferences can be explained by the model, hence the predictive power of the model was considered at a moderate level (Hair et al., 2019). Besides, the Q^2^ values (values of predictive relevance) were 0.30, 0.20, and 0.09 for cooperative, competitive, and individualistic learning preferences, respectively, indicating the model has sufficient predictive relevance (Shmueli et al., 2019).

In support of H1a, past cooperative learning experiences were positively associated with cooperative learning preferences (β = 0.54, *p* < 0.01), and negatively associated with individualistic learning preferences (β = −0.26, *p* < 0.01), supporting H1c. In line with H1d, past competitive learning experiences were positively related to competitive learning preferences (β = 0.23, *p* < 0.01).

SVOs were positively associated with I–C cultural identity (β = 0.28, *p* < 0.01), supporting H3 → H4 (mediating path), and also directly related to cooperative learning preferences (β = 0.14, *p* < 0.01), supporting H3a. I–C cultural identity was negatively associated with competitive (β = −0.55, *p* < 0.01) and individualistic learning preferences (β = −0.30, *p* < 0.01), consistent with H2b and H2c, but not significantly related to cooperative preferences (H2a not supported).

As predicted by H4b and H4c, I–C cultural identity mediated the relationships between SVO and both competitive (β = −0.15, *p* < 0.01) and individualistic learning preferences (β = −0.10, *p* < 0.01).

#### 4.2.4 Multigroup analysis

**Figure 4:**
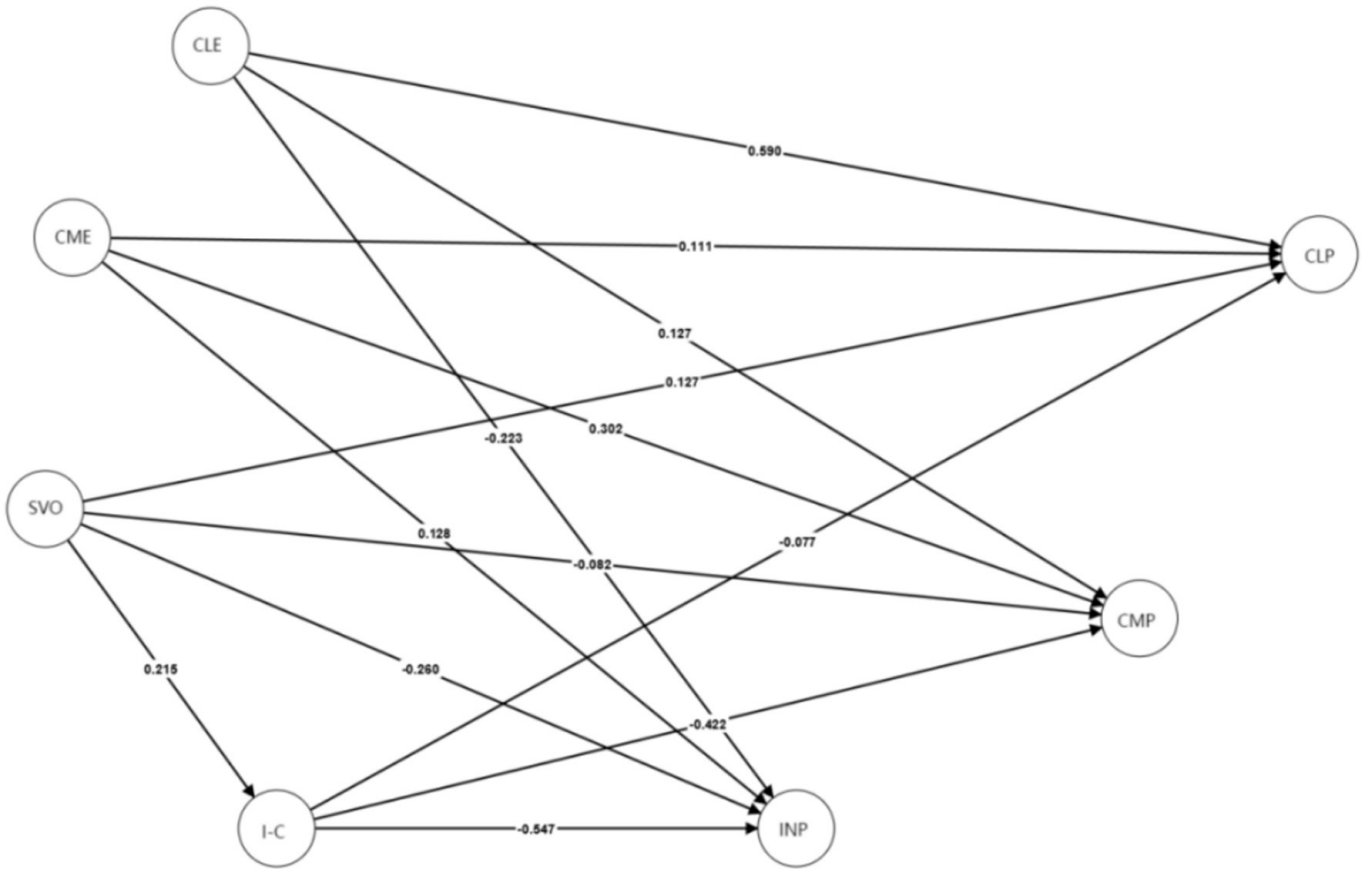
SEM diagram of previous learning experiences, SVOs, I-C cultural identity, and learning preferences for Chinese participants. Path coefficients are presented. CLE, cooperative learning experiences; CME, competitive learning experiences; SVO, social value orientation; I-C, I–C cultural identity; CLP, cooperative learning preference; CMP, competitive learning preference; INP, individualistic learning preference. The measurement part of the SEM model is not presented to make the diagram more readable.

**Figure 5:**
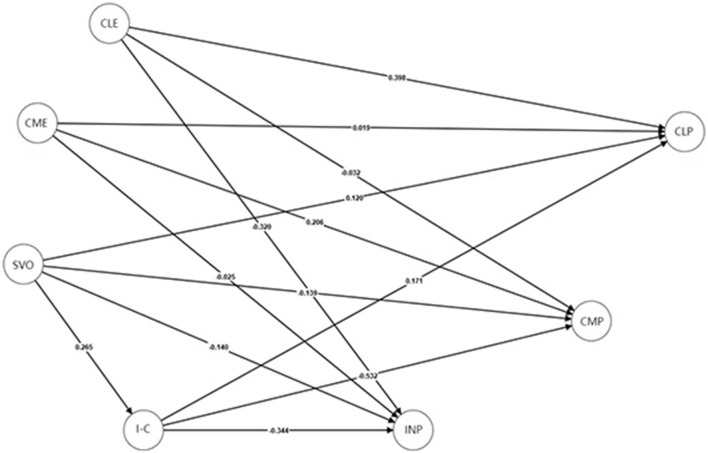
SEM diagram of previous learning experiences, SVOs, I-C cultural identity, and learning preferences for UK participants. Path coefficients are presented. CLE, cooperative learning experiences; CME, competitive learning experiences; SVO, social value orientation; I-C, I–C cultural identity; CLP, cooperative learning preference; CMP, competitive learning preference; INP, individualistic learning preference. The measurement part of the SEM model is not presented to make the diagram more readable.

To explore potential differences between the Chinese and UK participants, we conducted MGA PLS-SEM for further analysis (see Figures 4, 5 for China and UK, respectively). Table 4 summarizes the hypothesis testing results. Significant group differences (*p* < 0.05, two-tailed) emerged across several structural paths. Past cooperative learning experiences had a stronger effect on cooperative learning preferences in the Chinese sample (β = 0.59, *p* < 0.001) than in the UK sample (β = 0.40, *p* < 0.001). The path from cooperative learning experiences to competitive learning preferences was significant in the Chinese sample (β = 0.13, *p* < 0.05) but not in the UK sample. Both groups showed a significant negative relationship between cooperative learning experiences and individualistic preferences (CN: β = −0.22, *p* < 0.001; UK: β = −0.32, *p* < 0.001).

**Table 4:** Summary of hypothesis testing results of MGA-SEM.

**Hypothesis**	**Description**	**Supported**
H1a	Cooperative experiences → Cooperative learning preferences	Yes
H1a	Cooperative experiences → Competitive learning preferences (–)	Partially (only China)
H1a	Cooperative experiences → Individualistic learning preferences (–)	Yes
H1b	Competitive experiences → Competitive learning preferences	Yes
H1b	Competitive experiences → Cooperative learning preferences (–)	Partially (only China)
H1b	Competitive experiences → Individualistic learning preferences	Partially (only China)
H2	Collectivist identity → Cooperative learning preferences	Partially (only UK)
H2	Collectivist identity → Competitive learning preferences (–)	Yes
H2	Collectivist identity → Individualistic learning preferences (–)	Yes
H3	Prosocial SVO → Cooperative learning preferences	Yes
H3	Prosocial SVO → Competitive learning preferences (–)	Partially (only UK)
H3	Prosocial SVO → Individualistic learning preferences (–)	Yes
H4	I–C identity mediates SVO → Cooperative learning preferences	Partially (only UK)
H4	I–C identity mediates SVO → Competitive learning preferences	Yes
H4	I–C identity mediates SVO → Individualistic learning preferences	Yes

China refers to the Chinese sample; UK refers to the UK sample; *p* < 0.05, *p* < 0.01, *p* < 0.001.

Past competitive learning experiences were significantly associated with competitive preferences in both groups (CN: β = 0.30, *p* < 0.001; UK: β = 0.21, *p* < 0.01), and with individualistic preferences in China only (CN: β = 0.13, *p* < 0.05; UK: not significant).

Regarding SVO, it was positively related to cooperative preferences in both samples (CN: β = 0.13, *p* < 0.01; UK: β = 0.12, *p* < 0.05). SVO was negatively related to competitive preferences in the UK (β = – 0.14, *p* < 0.05), but not in China. It was negatively related to individualistic preferences in both groups (CN: β = −0.26, *p* < 0.001; UK: β = −0.14, *p* < 0.01).

I–C cultural identity was positively related to cooperative preferences in the UK only (β = 0.17, *p* < 0.05). It was negatively associated with competitive and individualistic preferences in both samples (competitive: CN: β = – 0.42, *p* < 0.001; UK: β = −0.53, *p* < 0.001; individualistic: CN: β = – 0.55, *p* < 0.001; UK: β = −0.34, *p* < 0.001).

For mediation effects, in China, SVO had direct effects on cooperative (β = 0.14, *p* < 0.01) and individualistic learning preferences (β = −0.14, *p* < 0.01), with partial mediation via I–C identity on individualistic preferences (β = −0.12, *p* < 0.05). In the UK, SVO had no direct effects on learning preferences. Instead, the effects were fully mediated by I–C identity: indirect effect on cooperative preferences (β = 0.05, *p* < 0.05), indirect effect on competitive preferences (β = – 0.14, *p* < 0.01), and indirect effect on individualistic preferences (β = −0.09, *p* < 0.01).

These findings highlight the differing mechanisms through which cultural identity influences learning preferences in distinct educational contexts.

## 5 Discussion

By examining students from China and the UK, the study highlights how educational practices and values systems inherited through cultural norms influence learning interactions and preferences in significant ways. These findings highlight the need for educational strategies that acknowledge and leverage these complex social dynamics.

### 5.1 Learning experiences and learning preferences

#### 5.1.1 Cooperative learning experiences

Chinese and UK students' cooperative learning experiences were positively related to cooperative learning preferences and negatively related to individualistic learning preferences, consistent with our hypotheses and findings of previous studies (Ryan and Wheeler, 1977; Johnson and Johnson, 2005; Choi et al., 2011). This observation also resonates with recent studies that students' prior experiences of cooperative work can shape students' perceptions of and preferences for cooperative learning (Bächtold et al., 2023; Stover and Holland, 2018). The theory of positive interdependence (Johnson and Johnson, 2005) suggests that cooperative learning environments, where success is mutually dependent, encourage promotive interactions among students. This environment facilitates a culture of mutual support, which is reflected in the students' preference for cooperative learning modalities. When positive interdependence is established in a learning environment, students may enjoy working together to maximize all the group members' learning outcomes mutually—for example, they may share their materials and support each other. Thus, we can infer from current findings that both Chinese and UK students' cooperation, mutual support, and helping behaviors in the classroom could have positive effects on their cooperative learning preferences and diminish individualistic intentions.

Interestingly, we observed that Chinese students' cooperative learning experiences were associated with competitive learning preferences, which appears counterintuitive at first glance. However, this pattern may reflect the complex nature of China's educational system, where cooperative activities are often embedded with a highly competitive academic culture. In classroom settings, students may be required to collaborate on assignments or projects while simultaneously being evaluated and ranked against their peers (Cortazzi and Jin, 1996). This coexistence of cooperative formats and performance-based assessments could foster a form of “instrumental cooperation” where students learn collaboratively not for mutual growth, but to gain a competitive edge (Cortazzi and Jin, 1996; Li, 2012). As a result, past experiences of working in groups may not diminish students' internalized competitiveness but enhance it in a subtle and socially adaptive way. The findings highlight the need to interpret educational constructs like cooperative and competitive learning within the cultural-institutional contexts, rather than as mutually exclusive categories.

Future research is needed to further explore this paradox, particularly through longitudinal or mixed-method approaches that can capture how competitive strategies may evolve within nominally cooperative learning environments, especially in education systems like China's where structural pressures may influence behavioral adaptation.

#### 5.2.2 Competitive learning experiences

Consistent with our hypotheses, the competitive learning experiences of both Chinese and UK student groups were found to be significantly positively associated with competitive learning preferences. As predicted by social interdependence theory, competitive learning experiences create a negative interdependence (Johnson and Johnson, 2009), where students are placed in win-lose situations. In such settings, students are compelled to compete against their peers to achieve personal academic goals, which naturally promotes a competitive learning orientation.

In both the Chinese and UK educational contexts, the pressure to excel in public examinations, such as China's Gaokao and the UK's A-levels and GCSEs, amplifies this competitive drive. These examinations not only play a pivotal role in determining future educational and career opportunities but also culturally promote a competitive ethos from a young age. This external competitiveness is internalized in students' learning preferences, with more exposure to competitive environments correlating with stronger competitive learning preferences.

Interestingly, the link between competitive learning experiences and individualistic learning preferences was notably stronger among Chinese students than their UK counterparts. This observation could be attributed to the highly exam-oriented nature of the Chinese educational system, where academic success is often pursued through individual efforts. Chinese educational practices typically emphasize individual performance, where students are frequently evaluated based on their solitary work during exams and are expected to complete assignments independently.

This distinction highlights a crucial aspect of how educational systems' structure and cultural expectations shape learning preferences. While both groups exhibit increased competitive learning preferences in response to competitive learning experiences, the Chinese system's emphasis on individual performance also fosters individualistic tendencies. This dual influence of competitive and individualistic predispositions in Chinese students suggests a complex interplay between cultural, educational, and psychological factors that merits further exploration.

Together, our findings highlight the significant role of undergraduates' past learning experiences in affecting current learning preferences. In a cross-cultural HE environment, educators, teachers and practitioners may need to bear in mind that students may enter the same classroom with different prior learning experiences, making them vary in perceptions of and preferences for different learning approaches. This might be particularly important when instructors planning to employ cooperative learning, since students prior learning experiences impact students' beliefs on the value and usefulness of cooperative learning as well as their willingness to cooperate (Bächtold et al., 2023), which further affects the successful implementation of such learning approach.

These findings highlight the need for educational policymakers and practitioners to consider the broader implications of competitive academic environments. While fostering a competitive spirit can drive academic excellence, it is also crucial to balance these environments with opportunities for collaborative and cooperative learning, especially in culturally diverse settings. Such balance could help mitigate the potential downsides of excessive competition, such as undue stress and isolation, while promoting a more holistic educational experience that values both individual achievement and collective progress.

#### 5.2.3 The impact of SVOs

Supporting our hypotheses, for both UK and Chinese groups, prosocials are more likely to prefer cooperative learning and less likely to prefer competitive or individualistic learning. Cooperative learning is characterized by situations where group members' collective efforts are essential to achieving shared group goals, fostering an environment where free-riding behaviors are discouraged (Johnson and Johnson, 2005). Prosocial students, as natural co-operators (Smeesters et al., 2003; Van Lange et al., 2007), demonstrate a stronger willingness to engage in cooperative learning due to their inclination to contribute to collective success. They also tend to avoid competitive learning, which fosters win-lose dynamics, and individualistic learning, which prioritizes self-interest over group collaboration.

Interestingly, for the Chinese group, SVOs were not significantly associated with competitive learning preferences. This may reflect the pervasive and normative nature of academic competition within China's education system, where competitive behaviors are structurally reinforced by examination pressures, ranking systems, and university entrance stakes (Liu and Littlewood, 1997; Zhu and Chang, 2019). In such contexts, even students with prosocial value orientations may adopt competitive learning strategies a pragmatic adaptation to structural demands, thereby weakening the predictive power of dispositional traits like SVOs.

For the UK group, the relationship between SVOs and learning preferences was fully mediated by I-C cultural identity, highlighting the critical role of cultural identity in shaping learning behaviors. SVOs influenced I-C cultural identity, which in turn informed learning preferences. In line with existing literature, I-C cultural identity reflects individuals' personal endorsement of individualist-collectivist values, which are influenced by SVOs (Moon et al., 2018) and predict learning preferences (Wagner, 1995). Our findings extend these theories by demonstrating that prosocial UK students are more likely to align with collectivist cultural values, which promote cooperative learning preferences.

In contrast, findings from the Chinese group revealed independent, direct effects of SVO and I-C cultural identity on learning preferences, with no mediation. Prosocial Chinese students demonstrated a preference for cooperative learning and a dislike for individualistic learning, driven by their inherent prosocial tendencies rather than an alignment with collectivist cultural values. This divergence may reflect the differing salience of cultural dimensions in Eastern vs. Western contexts. In Western societies, individualism-collectivism is often a more prominent cultural framework, amplifying the mediating role of I-C cultural identity. In Eastern societies, however, other cultural dimensions, such as power distance or uncertainty avoidance, may have stronger influences on behaviors and attitudes, potentially diminishing the role of I-C cultural identity.

Together, these findings suggest that the influence of SVOs on learning preferences is context-dependent, shaped by the interaction between individual value orientations and cultural frameworks. While I-C cultural identity serves as a critical mediator in the UK contexts, its relevance in Chinese contexts may be less pronounced. Future research should explore alternative cultural dimensions and their potential interactions with SVOs to provide a more comprehensive understanding of these dynamics across diverse cultural settings.

#### 5.2.4 The role of I-C cultural identity

For both UK and Chinese participants, a higher level of collectivist cultural identity was associated with lower preferences for competitive and individualistic learning, supporting our hypotheses. The findings align with prior research indicating that greater personal identification with collectivist cultural values is linked to a reduced inclination in competitive and individualistic learning approaches (Hall, 2017; Zhan et al., 2013). Students who prioritize group and interpersonal relationships (i.e., with stronger endorsement of collectivist identity) are likely to disfavor competitive and individualistic learning approaches that emphasize individual achievement over group cooperation and collective good. Collectivists often subordinate their personal interest in place of group values (Hofstede et al., 2010), making competitive and individualistic learning approaches less appealing due to their focus on dividual goals rather than group-oriented outcomes (Wagner, 1995).

Interestingly, the effect of collectivist cultural identity on cooperative learning preferences differed between the two samples. Among UK participants, a stronger personal endorsement of collectivist cultural value was positively associated with cooperative learning preferences, aligning with our hypotheses. This supports the notion that collectivist cultural identity, at both national and individual levels, is strongly aligned with group-oriented values over individualistic goals (Triandis, 2001; Hofstede et al., 2010). As a result, UK students with a collectivist cultural identity are more inclined to engage in cooperative learning, which fosters mutual support and collaboration within the classroom.

Contrary to our expectation, Chinese participants' collectivist cultural identity was not significantly related to cooperative learning preferences. This outcome might be explained by the distinct characteristics of collectivism in Chinese society. While collectivism is often associated with group values, in Chinese society, the primary collective unit is the family. Students with a strong collectivist identity may prioritize the collective good of their family over the classroom group. In Chinese culture, academic success is often viewed as an individual's contribution to the family's development (Annunziata et al., 2006). Within the highly competitive education system in China, where academic success is crucial for social mobility (Clark et al., 2007; Ross and Wang, 2010), collectivist cultural identity may not translate into a preference for cooperative learning in classroom settings. Instead, students may focus on achieving individual academic success to fulfill familial expectations and obligations.

The absence of a significant relationship between collectivist cultural identity and cooperative learning preferences among Chinese students suggests that collectivism may manifest differently in academic settings. It is plausible that in high-stakes educational environments, collectivist values prioritize collective achievement over cooperative learning behaviors within classroom interactions. These findings highlight the contextual variability in how collectivist cultural identity shapes learning preferences. While collectivism may generally discourage competitive and individualistic learning, its relationship with cooperative learning may depend on cultural characters, such as the specific collective units emphasized (e.g., family vs. classroom).

Future research should explore these dynamics further, examining how different cultural dimensions and contexts influence the interplay between collectivist cultural identity and learning preferences. Additionally, alternative cultural dimensions, such as power distance or uncertainty avoidance, may offer valuable insights into how cooperative learning behaviors are shaped in high-stakes educational environments like those in China.

In addition to the specific patterns observed across variables, it is important to consider the broader cultural contrasts that emerge from these findings. Compared to their UK counterparts, Chinese students' learning preferences were more directly shaped by their past cooperative and competitive learning experiences, suggesting a closer alignment with external and institutional learning environments. In contrast, UK students exhibited stronger indirect effects, with I-C identity mediating the influence of SVOs on learning preferences. This pattern indicates that Chinese students may be more influenced by normative expectations and external structures, whereas UK students' preferences may reflect more internalized orientations and culturally embedded self-perceptions. This contrast underscores the culturally contingent nature of both learning preferences and their psychological foundations.

## 6 Conclusion

This study investigates the interplay between past learning experiences, I-C cultural identity, and SVOs in shaping the cooperative, competitive, and individualistic learning preferences of Chinese and UK undergraduates. The findings reveal that these relationships vary significantly between the two groups, reflecting the distinct cultural and pedagogical environments of China and the UK. These results suggest that learning preferences are shaped by a socialization process embedded in unique cultural and educational contexts.

### 6.1 Theoretical contributions

This study contributes to the literature by integrating SVO, I-C cultural identity, and past learning experiences into an integrated model of learning preferences within a cross-cultural comparison. It advances current understanding of the complex dynamics between cultural identity and learning preferences, enriching theoretical perspectives on social interactions in education.

The findings extend social interdependence theory by highlighting how educational and cultural contexts influence cooperative, competitive, and individualistic learning preferences. Specifically, the study demonstrates the mediating role of I-C cultural identity in the relationship between SVO and learning preferences among UK students, highlighting the significant role of cultural norms and values in shaping educational interactions. This suggests how collective cultural narratives contribute to individual educational experiences.

The study further illustrates the differential effects of cultural and educational contexts on the relationship between SVOs, I-C cultural identity, and learning preferences. For example, while the mediation effect of I-C cultural identity is evident among UK students, it is less pronounced for Chinese students, suggesting cultural dimensions interact with personal and contextual factors in unique ways. This challenges the conventional dichotomy of individualism vs. collectivism and promotes an integrated approach to studying learning behaviors in cross-cultural contexts.

### 6.2 Practical implications

The findings have important implications for educational practice, particularly in diverse and globalized learning environments. The positive association between cooperative learning experiences and cooperative learning preferences emphasizes the need for educational strategies that foster collaborative learning environments. Such environments can enhance mutual support and group cohesion, which are particularly beneficial in diverse educational settings where students may have varied cultural and learning backgrounds. The insights into how competitive learning experiences influence learning preferences highlight the importance of balancing competitive tasks with cooperative learning opportunities. This balance can help mitigate the potential stress and isolation associated with highly competitive educational environments, especially among students who may prefer or benefit more from cooperative learning approaches.

Additionally, understanding how cultural identity influences learning preferences can inform the development of culturally responsive teaching strategies. Training programs for educators that integrate cultural awareness and pedagogical diversity can empower them to effectively manage multicultural classrooms, promoting inclusivity and engagement.

In globalized higher education, these insights are particularly relevant for institutions hosting diverse international cohorts, such as Chinese students in the UK. Equipping educators with the skills to adapt to students' cultural backgrounds, past learning experiences, and personal characteristics is vital for creating effective and inclusive learning environments.

## 7 Limitations and future directions

It is important to acknowledge the limitations of this study. First, our sample was non-randomly selected, meaning that it may not capture the full range of SVOs and learning preferences in both contexts. Second, it should be noted that the measure of I-C cultural identity in this study was based on Wagner's (1995) approach and, hence the cultural dimension of power distance was not included. Future research could consider incorporating this dimension by using a measure of I-C cultural identity that includes both power distance and I-C cultural dimension (such as Triandis's vertical vs. horizontal individualist-collectivist dimension). This could shed further light on the ways in which I-C cultural influences may vary among Chinese and UK students. Third, it is acknowledged that students' learning preferences may affect how they perceive learning environment. Hence, findings regarding possible cause-effect direction between learning experiences and learning preferences might be limited and conclusions drawn based on these current results need to be treated carefully. Further studies are necessary to identify possible conditions under which previous learning experiences lead to learning preferences and, in turn, how learning preferences result in perceiving experiences as cooperative or competitive.

There are several additional avenues for future research. First, I–C culture at the organizational level (i.e., classroom and school culture) may interact with the societal and individual culture, potentially affecting students' learning preferences and SVOs. Thus, future research incorporating I–C culture at the level of society, organizations, and individuals may contribute to a further understanding of these concepts. Second, we acknowledge the need to explore how learners with different SVOs and learning preferences may perceive the same context differently, as students' learning preferences could impact perceptions of the learning environment.

In summary, the study offers insights into the intricate dynamics among educational systems, cultural values, and personal character that contribute to the variations observed in how Chinese and UK students perceive and prefer different learning environments. As HE continues to globalize, these insights can guide the development of cross-cultural pedagogies that foster inclusive and effective learning environments, bridging cultural divides and enhancing student outcomes globally.

## Data Availability

The raw data supporting the conclusions of this article will be made available by the authors, without undue reservation.

## References

[B1] Annunziata D. Hogue A. Faw L. Liddle H. A. (2006). Family functioning and school success in at-risk, inner-city adolescents. J. Youth Adolesc. 35, 100–108. 10.1007/s10964-005-9016-3 21394228 PMC3050494

[B2] Bächtold M. Roca P. De Checchi K. (2023). Students' beliefs and attitudes towards cooperative learning, and their relationship to motivation and approach to learning. Stud. High. Educ. 48, 100–112. 10.1080/03075079.2022.2112028

[B3] Biggs J. B. (1991). Approaches to learning in secondary and tertiary students in Hong Kong: some comparative studies. Educ. Res. J. 6, 27–39.

[B4] Bogaert S. Boone C. Declerck C. (2008). Social value orientation and cooperation in social dilemmas: a review and conceptual model. Brit. J. Soc. Psychol. 47, 453–480. 10.1348/014466607X244970 17915044

[B5] Campbell J. Li M. (2008). Asian students' voices: An empirical study of Asian students' learning experiences at a New Zealand university. J. Stud. Int. Educ. 12, 375–396. 10.1177/1028315307299422

[B6] Cheah J. H. Thurasamy R. Memon M. A. Chuah F. Ting H. (2020). Multigroup analysis using smartpls: step-by-step guidelines for business research. Asian J. Bus. Res. 10, I–XIX. 10.14707/ajbr.200087

[B7] Chen-Xia X. J. Betancor V. Rodríguez-Gómez L. Rodríguez-Pérez A. (2023). Cultural variations in perceptions and reactions to social norm transgressions: a comparative study. Front. Psychol. 14:1243955. 10.3389/fpsyg.2023.1243955 37799515 PMC10548130

[B8] Choi J. Johnson D. W. Johnson R. (2011). Relationships among cooperative learning experiences, social interdependence, children's aggression, victimization, and prosocial behaviors. J. Appl. Soc. Psychol. 41, 976–1003. 10.1111/j.1559-1816.2011.00744.x

[B9] Clark J. Baker T. Li M. (2007). “Student success: bridging the gap for Chinese students in collaborative learning,” in Refereed Paper for the 18th ISANA Annual Conference (Glenelg: ISANA).

[B10] Cohen J. (2016). “A power primer,” in Methodological Issues and Strategies in Clinical Research, ed. Kazdin A. E. (Washington, DC: American Psychological Association), 279–284. 10.1037/14805-018

[B11] Cortazzi M. Jin L. X. (1996). “Cultures of Learning: Language Classrooms in China,” in Society and the Language Classroom, ed. Coleman H. (New York, NY: Cambridge University Press), 169–206.

[B12] Cubitt R. P. Starmer C. Sugden R. (1998). On the validity of the random lottery incentive system. Exp. Econ. 1, 115–131. 10.1023/A:1026435508449

[B13] Deutsch M. (1949). A theory of co-operation and competition. Hum. Relat. 2, 129–152. 10.1177/001872674900200204

[B14] Deutsch M. (1962). “Cooperation and trust: some theoretical notes,” in Nebraska Symposium on Motivation, ed. Jones M. R. (Lincoln, NE: University of Nebraska Press), 275–319.

[B15] Elliot A. J. Aldhobaiban N. Kobeisy A. Murayama K. Gocłowska M. A. Lichtenfeld S.. (2016). Linking social interdependence preferences to achievement goal adoption. Learn. Individ. Diff. 50, 291–295. 10.1016/j.lindif.2016.08.020

[B16] Field A. P. (2013). Discovering Statistics Using SPSS (and Sex and Drugs and Rock'n' Roll, 4th Edn. London: Sage.

[B17] Fraser B. J. (1998). Classroom environment instruments: development, validity and applications. Learn. Environ. Res. 1, 7–34. 10.1023/A:1009932514731

[B18] Fraser B. J. Anderson G. J. Walberg H. J. (1982). Assessment of Learning Environments: Manual for Learning Environment Inventory (LEI) and My Class Inventory (MCI), 3rd Edn. Perth, WA: Western Australian Institute of Technology.

[B19] Gächter S. Herrmann B. Thöni C. (2010). Culture and cooperation. Philos. Trans. R. Soc. B. Biol. Sci. 365, 2651–2661. 10.1098/rstb.2010.0135 20679109 PMC2936171

[B20] Gill S. (2007). Overseas students' intercultural adaptation as intercultural learning: A transformative framework. Compare 37, 167–183. 10.1080/03057920601165512

[B21] Gocłowska M. A. Aldhobaiban N. Elliot A. J. Murayama K. Kobeisy A. Abdelaziz A.. (2017). Temperament and self-based correlates of cooperative, competitive and individualistic learning preferences. Int. J. Psychol. 52, 180–188. 10.1002/ijop.12206 26314931

[B22] Grasha A. F. (2002). Teaching With Style: A Practical Guide to Enhancing Learning by Understanding Teaching and Learning Styles. Pittsburgh, PA: Alliance.

[B23] Hair Jr J. F. Sarstedt M. Ringle C. M. Gudergan S. P. (2017). Advanced Issues in Partial Least Squares Structural Equation Modeling. Thousand Oaks, CA: Sage Publications. 10.1007/978-3-319-05542-8_15-1

[B24] Hair J. F. Risher J. J. Sarstedt M. Ringle C. M. (2019). When to use and how to report the results of PLS-SEM. Eur. Bus. Rev. 31, 2–24. 10.1108/EBR-11-2018-0203

[B25] Hall G. C. N. (2017). Multicultural Psychology. New York, NY: Routledge.

[B26] Henseler J. Ringle C. M. Sarstedt M. (2015). A new criterion for assessing discriminant validity in variance-based structural equation modeling. J. Acad. Mark. Sci. 43, 115–135. 10.1007/s11747-014-0403-8

[B27] Henseler J. Ringle C. M. Sarstedt M. (2016). Testing measurement invariance of composites using partial least squares. Int. Mark. Rev. 33, 405–431. 10.1108/IMR-09-2014-0304

[B28] Henseler J. Ringle C. M. Sinkovics R. R. (2009). “The use of partial least squares path modeling in international marketing,” in New Challenges to International Marketing (Advances in International Marketing, Vol. 20), eds. Sinkovics R. R. Ghauri P. N. (Leeds: Emerald Group Publishing Limited), 277–319. 10.1108/S1474-7979(2009)0000020014

[B29] Higher Education Statistics Agency (2022). Higher Education Student Statistics. Cheltenham: Higher Education Statistics Agency.

[B30] Hofstede G. Hofstede G. J. Minkov M. (2010). Cultures and Organizations: Software of the Mind (3rd edn.). New York, NY: McGraw Hill.

[B31] Johnson D. W. Johnson R. T. (2005). “Cooperative learning, values, and culturally plural classrooms,” in Classroom Issues: Practice, Pedagogy and Curriculum (Education, Culture, and Values B Volume 3), eds. Leicester M. Modgil S. (Routledge), 29–47. 10.4324/9780203984109-9

[B32] Johnson D. W. Johnson R. T. (2009). An educational psychology success story: social interdependence theory and cooperative learning. Educ. Res. 38, 365–379. 10.3102/0013189X0933905732503387

[B33] Johnson D. W. Norem-Hebeisen A. A. (1979). A measure of cooperative, competitive, and individualistic attitudes. J. Soc. Psychol. 109, 253–261. 10.1080/00224545.1979.9924201

[B34] Kochanska G. (2002). Mutually responsive orientation between mothers and their young children: a context for the early development of conscience. Curr. Directions Psychol. Sci. 11, 191–195. 10.1111/1467-8721.001989084128

[B35] Kurzban R. Houser D. (2001). Individual differences in cooperation in a circular public goods game. Eur J Pers. 15, S37–S52. 10.1002/per.420

[B36] Lampridis E. Papastylianou D. (2017). Prosocial behavioral tendencies and orientation towards individualism-collectivism of Greek young adults. Int. J. Adolesc. Youth, 22, 268–282. 10.1080/02673843.2014.890114

[B37] Lee J. (2008). The effect of the background risk in a simple chance improving decision model. J. Risk Uncertainty 36, 19–41. 10.1007/s11166-007-9028-3

[B38] Li J. (2012). Cultural Foundations of Learning: East and West. Cambridge: Cambridge University Press. 10.1017/CBO9781139028400

[B39] Liu N. F. Littlewood W. (1997). Why do many students appear reluctant to participate in classroom learning discourse?. System 25, 371–384. 10.1016/S0346-251X(97)00029-8

[B40] Ma C. Shen Y. (2024). Understanding food waste sorting behavior in institutional food services: an integrated psychological framework. J. Environ. Manage. 360:121215. 10.1016/j.jenvman.2024.121215 38781879

[B41] McClintock C. G. (1978). Social values: their definition, measurement, and development. J. Res. Dev. Educ. 12, 121–137.

[B42] Messick D. M. McClintock C. G. (1968). Motivational bases of choice in experimental games. J. Exp. Soc. Psychol. 4, 1–25. 10.1016/0022-1031(68)90046-222027427

[B43] Montgomery S. M. Groat L. N. (1998). Student Learning Styles and Their Implications for Teaching (CRLT Occasional Paper No. 10). Ann Arbor, MI: Center for Research on Learning and Teaching, University of Michigan.

[B44] Moon C. Travaglino G. A. Uskul A. K. (2018). Social value orientation and endorsement of horizontal and vertical individualism and collectivism: an exploratory study comparing individuals from North America and South Korea. Front. Psychol. 9:2262. 10.3389/fpsyg.2018.02262 30515124 PMC6255969

[B45] Murphy R. O. Ackermann K. A. (2014). Social value orientation: Theoretical and measurement issues in the study of social preferences. Pers. Soc. Psychol. Rev. 18, 13–41. 10.1177/1088868313501745 24065346

[B46] Murphy R. O. Ackermann K. A. Handgraaf M. (2011). Measuring social value orientation. Judgm. Decis. Mak. 6, 771–781. 10.1017/S1930297500004204

[B47] Nguyen P. Terlouw C. Pilot A. (2006). Culturally appropriate pedagogy: the case of group learning in a Confucian Heritage Culture context. Intercult. Educ. 17, 1–19. 10.1080/14675980500502172

[B48] Nguyen-Phuong-Mai M. (2019). Culturally appropriate face strategies in cooperative learning with insight from cultural neuroscience. Comp. Educ. 55, 66–96. 10.1080/03050068.2018.1541664

[B49] OECD (2014). TALIS 2013 Results: An International Perspective on Teaching and Learning. Paris: OECD. 10.1787/9789264196261-en

[B50] Owens L. Barnes J. (1982). The relationships between cooperative, competitive, and individualized learning preferences and students' perceptions of classroom learning atmosphere. Am. Educ. Res. J. 19, 182–200. 10.3102/0002831201900218238293548

[B51] Pan Z. Cutumisu M. (2024). Using machine learning to predict UK and Japanese secondary students' life satisfaction in PISA 2018. Brit. J. Educ. Psychol. 94, 474–498. 10.1111/bjep.12657 38129097

[B52] Ping W. (2010). A case study of an in-class silent postgraduate Chinese student in London Metropolitan University: a journey of learning. TESOL J. 2, 207–214.

[B53] Qu X. Cross B. (2024). UDL for inclusive higher education-What makes group work effective for diverse international students in UK?. Int. J. Educ. Res. 123:102277. 10.1016/j.ijer.2023.102277

[B54] Ringle C. M. Wende S. Becker J. (2023). SmartPLS (Version 4). Bönningstedt (Hamburg): SmartPLS, GmbH.

[B55] Ross H. Wang Y. (2010). The college entrance examination in China: an overview of its social-cultural foundations, existing problems, and consequences: guest editors' introduction. Chin. Educ. Soc. 43, 3–10. 10.2753/CED1061-1932430400

[B56] Ryan F. L. Wheeler R. (1977). The effects of cooperative and competitive background experiences of students on the play of a simulation game. J. Educ. Res. 70, 295–299. 10.1080/00220671.1977.10885009

[B57] Sarstedt M. Hair J. F. Ringle C. M. Thiele K. O. Gudergan S. P. (2016). Estimation issues with PLS and CBSEM: where the bias lies! J. Bus. Res. 69, 3998–4010. 10.1016/j.jbusres.2016.06.007

[B58] Shmueli G. Sarstedt M. Hair J. F. Cheah J. H. Ting H. Vaithilingam S.. (2019). Predictive model assessment in PLS SEM: Guidelines for using PLSpredict. Eur. J. Mark. 53, 2322–2347. 10.1108/EJM-02-2019-0189

[B59] Slavin R. E. (2014). Cooperative learning and academic achievement: why does groupwork? Ann. Psychol. 30, 785–791. 10.6018/analesps.30.3.20120129329024

[B60] Smeesters D. Warlop L. Van Avermaet E. Corneille O. Yzerbyt V. (2003). Do not prime hawks with doves: the interplay of construct activation and consistency of social value orientation on cooperative behavior. J. Pers. Soc. Psychol. 84, 972–987. 10.1037/0022-3514.84.5.972 12757142

[B61] Stover S. Holland C. (2018). Student resistance to collaborative learning. IJ-SoTL 12:8. 10.20429/ijsotl.2018.120208

[B62] Taras V. Rowney J. Steel P. (2010). Examining the impact of culture's consequences: a three-decade, multilevel, meta-analytic review of Hofstede's cultural value dimensions. J. Appl. Psychol. 95, 405–439. 10.1037/a0018938 20476824

[B63] Triandis H. C. (2001). Individualism-collectivism and personality. J. Pers. 69, 907–924. 10.1111/1467-6494.696169 11767823

[B64] Trompenaars F. (1993). Riding the Waves of Culture: Understanding Cultural Diversity in Business. London: Nicholas Brealey.

[B65] Van Lange P. A. Bekkers R. Schuyt T. N. Vugt M. V. (2007). From games to giving: social value orientation predicts donations to noble causes. Basic Appl. Soc. Psychol. 29, 375–384. 10.1080/01973530701665223

[B66] Van Lange P. A. De Bruin E. Otten W. Joireman J. A. (1997). Development of prosocial, individualistic, and competitive orientations: theory and preliminary evidence. J. Pers. Soc. Psychol. 73, 733–46. 10.1037/0022-3514.73.4.733 9325591

[B67] Wagner III J. A. (1995). Studies of individualism-collectivism: effects on cooperation in groups. Acad. Manage. J. 38, 152–173. 10.2307/256731

[B68] Wen J. Huang S. S. Teo S. (2023). Effect of empowering leadership on work engagement via psychological empowerment: moderation of cultural orientation. J. Hosp. Tour. Manage. 54, 88–97. 10.1016/j.jhtm.2022.12.012

[B69] Zhan G. Q. Moodie D. Yanmin S. Wang B. (2013). An investigation of college students' learning styles in the US and China. J. Learn. High. Educ. 9, 169–178.

[B70] Zhang J. Han T. (2023). Individualism and collectivism orientation and the correlates among Chinese college students. Curr. Psychol. 42, 3811–3821. 10.1007/s12144-021-01735-2

[B71] Zhang Y. Zhu Y. Huang Z. Long L. (2024). Compensatory effects of loneliness: how and when does workplace loneliness promote employees unethical pro-organizational behavior. Curr. Psychol. 43, 24308–24319. 10.1007/s12144-024-06103-4

[B72] Zhu N. Chang L. (2019). “Education and parenting in China,” in School Systems, Parent Behavior, and Academic Achievement, Vol. 3, eds. Sorbring E. Lansford J. E. (Cham: Springer), 17–29. 10.1007/978-3-030-28277-6_2

