# Correction: Exploring influences of past learning experiences, individualist-collectivist cultural identity and social value orientations on Chinese and UK undergraduates' learning preferences

**DOI:** 10.3389/fpsyg.2025.1654666

**Published:** 2025-07-18

**Authors:** Chengcheng Ma, Shelley McKeown, Jo Rose

**Affiliations:** ^1^Institute for Intelligent Society Governance, Tsinghua University, Beijing, China; ^2^School of Education, University of Bristol, Bristol, United Kingdom; ^3^Department of Experimental Psychology, University of Oxford, Oxford, United Kingdom

**Keywords:** learning preferences, social value orientation, individualist-collectivist culture, learning experiences, cooperative learning

In the published article, there was an error regarding the affiliations for author Shelley McKeown. As well as having affiliation 2, “School of Education, University of Bristol, Bristol, United Kingdom”, they should also have new affiliation 3, “Department of Experimental Psychology, University of Oxford, Oxford, United Kingdom”.

The original version of this article has been updated.

